# Discrepancies between Antimullerian Hormone and Follicle Stimulating Hormone in Assisted Reproduction

**DOI:** 10.1155/2013/383278

**Published:** 2013-12-18

**Authors:** Munawar Hussain, David Cahill, Valentine Akande, Uma Gordon

**Affiliations:** ^1^St. Michael's Hospital, University Hospital Bristol NHS Foundation Trust, Southwell Street Bristol, BS2 8EG, UK; ^2^University of Bristol, St. Michael's Hospital, Bristol BS2 8EG, UK; ^3^Southmead Hospital, North Bristol NHS Trust & Bristol Centre for Reproductive Medicine Bristol, Southmead Road, Westbury-on-Trym, Bristol BS10 5NB, UK

## Abstract

Data from 107 women undergoing their first IVF/ICSI were analyzed. Relationships between antimullerian hormone (AMH) and follicle stimulating hormone (FSH) were analyzed after dividing patients into four groups according to AMH/FSH levels. Concordance was noted in 57% of women (both AMH/FSH either normal or abnormal) while 43%of women had discordant values (AMH/FSH one hormone normal and the other abnormal). Group 1 (AMH and FSH in normal range) and group 2 (normal AMH and high FSH) were younger compared to group 3 (low AMH and normal FSH) and group 4 (both AMH/FSH abnormal). Group 1 showing the best oocyte yield was compared to the remaining three groups. Groups 3 and 4 required higher dose of gonadotrophins for controlled ovarian hyperstimulation showing their low ovarian reserve. There was no difference in cycle cancellation, clinical pregnancy, and live birth/ongoing pregnancy rate in all groups. These tests are useful to predict ovarian response but whether AMH is a substantially better predictor is not yet established.

## 1. Introduction

Correct assessment of ovarian reserve and prediction of ovarian response to gonadotrophin stimulation are important for patients undergoing assisted reproduction treatment (ART) and are a core issue in modern fertility management [1]. Over the last two decades, several ovarian reserve tests have been proposed to predict ovarian response and ART outcome and all the available ovarian reserve testing methods have limitations [2]. Even the best available ovarian reserve test is associated with 10–20% false positive results [3].

Basal follicle stimulating hormone (FSH) is still the most commonly used ovarian reserve test though its reliability as an ovarian reserve test is weak [4]. Elevated day-3 FSH level is associated with poor response to gonadotrophin stimulation [5] and significantly lower chances of pregnancy [6]. It can only predict ovarian response and chances of pregnancy only at high threshold of values [1, 4, 7]. Using FSH leads to the inconvenience of menstrual cycle day 2–5 testing and is associated with significant inter- and intracycle variability [8].

Antimullerian hormone (AMH) is considered a specific marker of ovarian response to gonadotrophins [3]. A single random measurement of AMH has 80% sensitivity and 93% specificity to predict poor ovarian response [9]. Its levels correlate with the number of oocytes retrieved and treatment can be individualized for optimal cycle [3, 10–14]. As opposed to FSH, it can be tested on any day of the menstrual cycle as opposed to FSH which needs to be measured during early follicular phase. However, it has been shown that AMH values can vary between different blood samples of the same patient during the same menstrual cycle especially in young patients [15, 16].

Both AMH and FSH are still in use as ovarian reserve tests. Despite statistical congruity between FSH and AMH [17], many individual patients have discordant values which makes it difficult to counsel patients regarding their true ovarian reserve and cycle outcome [18–20]. Moreover, controversies regarding different assays [21], pregnancies even at undetectable levels [22], and intracycle variations in AMH levels [16] have left some unanswered questions about the possible role of AMH in assisted reproduction.

FSH and AMH are two different hormones and predict ovarian reserve at two different stages of follicular development. FSH levels reflect antral and postantral follicular development while AMH values are representative of postprimordial preantral follicular pool [18]. Despite the use of both these hormones in parallel to determine ovarian reserve, there is not much literature [18] about the frequency of discordance and concordance between them and its clinical significance. We therefore conducted this study to determine the frequency of concordance and discordance between AMH and FSH levels and what the ART cycle outcome was in women with concordant and discordant AMH/FSH values.

## 2. Material and Methods

This cohort study was conducted at Bristol Centre for Reproductive Medicine (BCRM) from September 2010 to May 2012. Women undergoing their first IVF/ICSI cycle who had their ovarian reserve assessed by both Gen-2 AMH assay and FSH were included. All patients gave written consent for surgical procedures and anonymised use of laboratory and clinical data for quality and research purposes.

### 2.1. Hormone Assays

FSH and AMH were tested within 6 months of undergoing ART. FSH was tested on days 1–5 of the menstrual cycle and in case of more than one available result, the highest level of FSH was selected. FSH analysis was performed on a Siemens Immulite 2000 (Siemens Healthcare Diagnostics Inc., Tarrytown, NY) by a chemiluminescent immunoassay (lower limit of detection 0.1 IU/L, interassay CV of 6.8, 5.4, and 4.3% at levels of 6.6, 14.9, and 38 IU/L). AMH was assayed through a commercially available assay on any day of the menstrual cycle at patient's convenience. AMH levels were measured using the Generation II AMH (Gen II AMH assay) enzyme-linked immunosorbent assay kit (Beckman Coulter, Inc., USA). Intra- and inter-assay coefficients of variation (CV) were <6 and <10%, respectively, lower detection limit was at 0.08 ng/mL, and linearity was up to 21 ng/mL for AMH. AMH levels were reported as pmol/L (conversion factor: 1 ng/mL = 7.14 pmol/L).

### 2.2. Controlled Ovarian Hyperstimulation (COH) Protocol

Our COH protocol has been explained in detail [23]. In brief, all patients underwent pituitary downregulation with long protocol of gonadotrophin releasing hormone agonist nasal spray (Suprefact; Aventis Pharma, Kent, UK). Individualized doses of FSH were used according to patient's age, basal FSH, or AMH levels. Patients <35 years of age, with optimal ovarian reserve confirmed by normal FSH/AMH, levels were started on 150 IU of gonadotrophins and patients ≥35 years, with high FSH/low AMH, were started on 300 IU of gonadotrophins. Transvaginal ultrasound guided oocyte retrieval was performed under sedation, 34–36 hours after 6500 IU injection of recombinant human chorionic gonadotrophin (Ovitrelle; Merck Serono, UK). For luteal phase support, 400 mg Progesterone vaginal pessaries (Cyclogest; Shire Pharmaceuticals Ltd., Basingstoke, Hants, UK) 12 hourly were used. As per local policy, single embryo or blastocyst was transferred by soft embryo transfer catheter (Sydney Cook, Limerick, Ireland). Urinary pregnancy test was performed two weeks after embryo transfer if no period ensued. Early pregnancy ultrasound scan was carried out at 6-7 weeks of gestation to confirm ongoing pregnancy.

### 2.3. Statistical Analysis

For statistical evaluation patients were divided into four groups according to their AMH and FSH levels. AMH level of <5 pmol/L was considered reduced and above 5 pmol/L was considered normal [12]. FSH level of ≤10 IU/L was considered normal and above 10 IU/L was considered as high [24]. Group 1 included women with normal AMH and normal FSH levels (nAMH/nFSH), group 2 included women with normal AMH and high FSH levels (nAMH/hFSH), group 3 included women with low AMH and normal FSH levels (lAMH/nFSH), and group 4 included women with low AMH and high FSH levels (lAMH/hFSH).

Statistical analyses were performed by Stats Direct (Stats Direct, Sale, UK). Nonparametric variables were expressed as median (interquartile range) and compared by Kruskal-Wallis test. Categorical variables were expressed as numbers with proportions (%) and compared by Fisher's exact test. A *P* value < 0.05 was considered statistically significant.

## 3. Results

A total of 107 women who reached their first IVF/ICSI cycle were included in the final analysis. Four cycles (4%) were cancelled due to poor ovarian response. Of the remaining 103 cycles, there was complete failure of fertilization in 7 cycles and 96 (90%) patients reached embryo transfer stage per started cycle.

Sixty-one women (57%) had concordant (groups 1 and 4) AMH and FSH values; both these hormones were either in normal or abnormal range. Group 1 included 37 women (35%) with both normal AMH and FSH levels (nAMH/nFSH) and group 4 included 24 women (22%) with low AMH and high FSH levels (lAMH/hFSH). However, forty-six women (43%) had discordant AMH and FSH values. In this category, group 2 included 7 (7%) women who had normal AMH but high FSH levels (nAMH/hFSH) and group 3 included 39 (36%) women who had low AMH but normal FSH levels (lAMH/nFSH).

Table 1 shows the demographic information and outcome of COH in the four groups. Group 1 showed the highest oocyte yield, but the number decreased significantly from group 1 to group 4. No differences were noted in the number of oocytes retrieved in groups 2, 3, and 4 and in the total days of stimulation for all groups though higher dose of gonadotrophin was used in groups 3 and 4 as compared with groups 1 and 2.

Table 2 summaries the cycle outcome. There was no statistical difference in fertilization, clinical pregnancy, and live birth/ongoing pregnancy rates in the four groups.

## 4. Discussion

Correct assessment of ovarian reserve is important for patients undergoing assisted reproduction treatment. Historically FSH has been the main tool but recently AMH has gained popularity and is considered more specific in predicting ovarian reserve and ovarian response to gonadotrophins stimulation [14]. Both FSH and AMH predict ovarian reserve independently and have been shown to correlate well [17]. When AMH and FSH are used in parallel, significant proportion of patients will have discordant values of these two hormones [18, 19]. Until further AMH outcome data are available, should both FSH and AMH be assayed in parallel to have the greatest likelihood of detecting reduced ovarian reserve [18, 25–28]?

Our data have shown that while AMH and FSH are often concordant, there is a high level of discordance (43% of cases) consistent with the published literature [18, 19]. It has been suggested that discordance between AMH and FSH is common and testing both FSH and AMH in parallel will improve ovarian reserve assessment in patients undergoing ART [20, 25–28]. The possible causes of discordance are either laboratory related variation in hormone levels [19] or intra-/intercycle variability of AMH and FSH. FSH has significant inter-cycle variability [8] and is highly dependent on the type of FSH analyzer used. Moreover, AMH has been shown to have false positive results in 10–15% cases [3] which can be another possible cause of discordance. In our study although FSH measurement was timed on days 2–5 of menstrual cycle it was not confirmed by concomitant estradiol testing. This might have led to false negative FSH values and this could have led to discordance between FSH and AMH.

AMH, in contrast to FSH, has been reliably shown to be quite stable during the whole menstrual cycle [8, 29]. However, recently there have been reports of significant intracycle variations in AMH values [15, 16]. AMH Generation II (AMH Gen II) assay has been reported to give more uniform and consistent AMH values as compared to Generation I (AMH Gen I) assays. However, even AMH Gen II assay can display appreciable variability, which may be explained by sample instability. AMH Gen II values were >20% lower than those generated using the DSL assay instead of the 40% increase predicted by the kit manufacturer. Some storage or assay conditions may promote instability and this may be more pronounced with the Gen-II assay [30].

Normal AMH values may be more important than normal FSH values to predict oocyte yield and cycle cancellation rate. Adding FSH to AMH did not have a better oocyte yield in situations when the AMH was normal. In contrast, oocyte yield was significantly reduced even when FSH was reported normal [18, 20, 31]. Our data suggest that although there was no difference in the cycle cancellation rate between the groups, women with low AMH levels (groups 3 and 4) required significantly higher gonadotrophin doses for COH. Oocyte yield differed depending on the group observed: it was reduced from group 1 to 4, and oocyte numbers retrieved in groups 2, 3, and 4 were lower compared with group 1. Women with normal AMH values (group 2) might be expected to produce more oocytes compared with groups 3 or 4 but did not. There is a possibility that normal AMH levels in this group were false positive which is noted in 10–15% of cases [3] and high levels of FSH were representative of their reduced ovarian reserve leading to retrieval of less number of eggs. High FSH levels therefore still have value in predicting poor ovarian response, despite normal AMH. Groups 3 and 4 did have reduced oocyte numbers: this was a predicted finding with the reduced AMH levels in both these groups. In group 3, normal FSH levels were noted and this association might be related to the previously observed FSH cyclical fluctuation [7], a major limitation with FSH measurement. AMH levels begin to fall earlier than any appreciable rise in FSH [32] and the normal FSH levels in group 3 will of course provide false reassurance to the clinician and patient in the absence of an AMH.

Analyzing the groups by age would not have been meaningful due to the small numbers. Others have shown that both young patients with reduced ovarian reserve [33] or older women with normal AMH can have good oocyte yield [34, 35]. However, these results are somewhat more difficult to interpret as supplements such as dihydroepiandrosterone (DHEA) were used with good effect [16, 36].

In the present study, clinical pregnancy and live birth/ongoing pregnancy rates were comparable in all groups. There was a trend towards lower clinical pregnancy and live birth/ongoing pregnancy rates in groups 3 and 4 (both with low AMH) which might ultimately be important in clinical settings. AMH is reported as a quantitative ovarian reserve marker; however, its role in predicting more qualitative aspects of ART is not clear [3]. Positive correlations between serum AMH concentration and live birth after ART may be primarily due to its relation with the number of retrieved oocytes [11] leading to the apparent low clinical pregnancy/live birth rates observed in groups 3 and 4. Although some authors have found a positive correlation between AMH values and oocytes quality [37, 38] others [39–41] failed to show this relationship. Moreover, the relationship between serum AMH concentration and live birth is not established and it is not an independent marker of pregnancy outcome [3, 11, 42]. Women with high FSH have reasonable chance of achieving pregnancy providing they reach embryo transfer.

This study is limited by its retrospective nature and the small number of patients, making it difficult to generalize results. Moreover, analysis by age could not meaningfully be undertaken. However, we believe this paper is the first to investigate concordance between AMH and FSH using Gen II AMH assays.

## 5. Conclusion

Women with both normal AMH and FSH values have the best oocyte yield as compared to women who have either one or both of these hormones in the abnormal range. Significant discordance between AMH and FSH may assist counseling and prediction of cycle outcome in patients undergoing ART treatments, but the role of AMH in predicting ovarian response is not yet fully understood.

## Figures and Tables

**Table 1:** Comparison of the demographics and cycle outcome.

Demographics and cycle outcome	Group 1 (nAMH/nFSH) *n* = 37	Group 2 (nAMH/hFSH) *n* = 7	Group 3 (lAMH/nFSH) *n* = 39	Group 4 (lAMH/hFSH) *n* = 24	*P* value
*Age (years)	34 (32–38)	33 (32-33)	37 (34–39)	37 (35–39)	0.0005
*BMI (kg/m^2^)	25 (23–27)	23 (21–28)	22 (21–23)	24 (23–28)	0.27
*AMH (pmol/L)	13.1 (7.38–27.4)	8.8 (6.8–13.1)	2.2 (0.7–3.9)	1.4 (0.4–3.1)	<0.0001
*FSH (IU/L)	7.1 (6–8.2)	12.54 (10.7–18)	8 (6.4–10)	12 (11.2–32.9)	<0.0001
*COH (days)	14 (14-15)	14 (13–15)	14 (13–16)	15 (14–16)	0.87
*FSH dose (IU)	3600 (2250–4200)	3300 (2250–4299)	4200 (3000–4500)	4500 (4200–4800)	0.0008
*No. of oocytes retrieved	7 (5–10)	3 (2–7)	4 (3–6)	4 (2–6)	0.0023
**Cycles cancelled (%)	1 (2%)	0	2 (5%)	1 (4%)	0.90

BMI: body mass index, COH: controlled ovarian hyperstimulation, and OR: ovarian reserve. *Values presented as median with interquartile range in parenthesis. **Numbers with percentages in parenthesis.

**Table 2:** Comparison of failed fertilization, clinical pregnancy, miscarriage, and live birth/ongoing pregnancy rates.

Cycle outcome	Group 1 (nAMH/nFSH) *n* = 37	Group 2 (nAMH/hFSH) *n* = 7	Group 3 (lAMH/nFSH) *n* = 39	Group 4 (lAMH/hFSH) *n* = 24	*P* value
Failed fertilization (%)	2 (5)	0	2 (5)	3 (12)	0.55
Clinical pregnancy rate (%)	9 (24)	2 (28)	6 (15)	4 (16)	0.70
Miscarriage rate (%)	0	0	1 (17)	0	<0.0001
Live birth/ongoing pregnancy rate (%)	9 (24)	2 (28)	5 (12)	4 (16)	0.54
